# GeneMiner: A Classification Approach for Detection of XSS Attacks on Web Services

**DOI:** 10.1155/2022/3675821

**Published:** 2022-06-25

**Authors:** Charu Gupta, Rakesh Kumar Singh, Amar Kumar Mohapatra

**Affiliations:** Department of IT, Indira Gandhi Delhi Technical University for Women, Delhi, India

## Abstract

According to OWASP 2021, cross-site scripting (XSS) attacks are increasing through specially crafted XML documents. The attacker injects a malicious payload with a new pattern and combination of scripts, functions, and tags that deceits the existing security mechanisms in web services. This paper proposes an approach, GeneMiner, encompassing GeneMiner-E to extract new features and GeneMiner-C for classification of input payloads as malicious and nonmalicious. The proposed approach evolves itself to the changing patterns of attack payloads and identifies adversarial XSS attacks. The experiments have been conducted by collecting data from open source and generating various combinations of scripts, functions, and tags using an incremental genetic algorithm. The experimental results show that the proposed approach effectively detects newly crafted malicious XSS payloads with an accuracy of 98.5%, which is better than the existing classification techniques. The approach learns variations in the existing attack sample space and identifies the new attack payloads with reduced efforts.

## 1. Introduction

**Figure 1:**
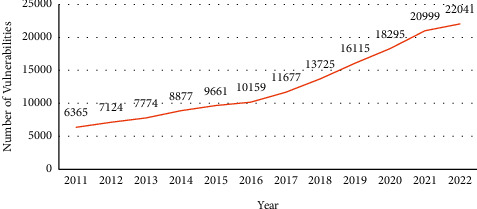
Increasing trend of XSS attacks.

Web services provide a solution for information exchange over web applications developed on varied platforms [1, 2] and facilitate integration and interoperability among heterogeneous software applications using XML (eXtensible Markup Language) [3, 4]. Many available protocols and standards provide security to web services, but attacks on web services are continuously increasing [1]. According to OWASP [5], 94% of web applications are tested for one or more forms of injection attacks. The number of cross-site script (XSS) injection attacks has increased from 470 in 2011 to 22,000 in April 2022 [6], as shown in Figure 1. The injection attacks occur due to the attacker's insertion of malicious characters and strings in XML documents [1, 7]. The attacker crafts the payload in such a manner to bypass the existing filters. The injection attacks have caused disclosure, distortion, disruption, and destruction of sensitive information and defacement of websites [1]. The impact of injection attacks is scored 7.25 on an average on a scale of 10 in terms of severity [8].

Adversarial attack payload refers to a newer combination of malicious characters, strings, tags, and scripts. Attackers deceive the available security standards, mitigation approaches, security testing, filters and firewalls, intrusion detection, and prevention systems by injecting a malicious adversarial payload. Suppose an algorithm is trained to detect payloads consisting of a script and alert tags. In that case, malicious attack payloads easily deceive the trained algorithm by using newer functions and obfuscation techniques. For example, techniques trained for detecting the payload <script> alert(“XSS”) </script> are easily deceived by newer payload onload = alert(/hacked/) by using *onload* and obfuscation of *alert*.

Machine learning (ML) [9–13] and neural network (NN) [14–17] techniques have recently been used to detect XSS vulnerability in source code. Genetic algorithms and fuzzy inference are combined with ML and NN to generate a large number of permutations and combinations of payloads using the existing datasets to mitigate the newer adversarial attacks [18–21]. Existing algorithms are limited by training on a fixed number of features and datasets. Further, the authors observed the following issues in the existing techniques and approaches:

1. Existing models are nonadaptive and do not fit into a dynamic and ever-changing real-world environment where attackers generate malicious payloads, each with a different combination and unique enough to escape the filters. The malicious attack payloads have a mix of encoded characters, case mixtures, recursively nested keywords, blank spaces, and tags to bypass filters.
2. With the ever-changing diversity in injection payloads and increase in feature set, the training algorithm becomes multidimensional, increasing the computational complexity manifolds.

In this paper, the authors propose an approach, GeneMiner, that identifies known XSS attacks and can detect the unknown and newer attack patterns. The proposed approach has utilized the properties of the incremental genetic algorithm to detect the ever-evolving heterogeneous and changing XSS payloads. The key contributions and innovations of the paper are as follows:

1. The proposed approach evolves to detect the adversarial malicious payloads consisting of newer functions, features, combinations, encoded characters, and obfuscation techniques.
2. The approach is optimized to search in the large multidimensional repositories by reducing it to single-dimensional search space.
3. The approach will learn variations in the existing attack sample space and evolve to the new attack environment consisting of a newer feature set with reduced efforts.
4. The authors conducted experiments with 1,60,264 records for performance evaluation of the newly added feature including comparison with other widely used machine learning and neural network models.

The rest of the paper is organized as follows. In Section 2, related work is discussed.  Section 3 contains the preliminaries to understand the proposed approach. In Section 4, the proposed XSS attack detection model—GeneMiner—is explained. The results of the experiments and their discussions are given in Section 5. In Section 6, conclusions and future work are discussed.

## 2. Related Work

NVD [8], Common Weakness Enumeration [22], and Common Attack Pattern Enumeration Catalog [23] enlisted that the most common attacks are XSS attacks via crafted XML documents [8]. XML cross-site scripting attack is carried out by manipulating the logic of XML-based web services. Hyper Text Markup Language (HTML) tags, JavaScript functions, and other characters are injected into XML messages to create an XSS attack. XSS attacks are also made by injecting parts of the attack vector in two or more input fields such that it bypasses the filter [24]. These XSS attacks on web services are capable of triggering phishing attacks, cookie theft, denial of service (DoS) attacks, distributed denial of service (DDoS) attacks, XSS worms, and browser screenshot theft.

### 2.1. Detection of XSS Attacks Using Neural Network

Fang et al. [14] and Lei et al. [15] extracted six categories of features using word2vec and trained the model using LSTM recurrent neural network. The method DeepXSS [14] included a decoder to detect obfuscated malicious payloads. The network detection model proposed by [15] was based on long short-term memory (LSTM) to extract abstract features of XSS attacks. Lei et al. [15] added a hidden layer of the attention mechanism to extract more relevant information to improve the classification using the recurrent neural network.

Melicher et al. [17] trained deep neural network using taint tracking methods to predict the vulnerability of payloads by analyzing JavaScript functions. Liu et al. proposed an approach GraphXSS, for the detection of XSS attacks, which converted an XSS payload into a graph of interconnected words and characters. Fang et al. [20] combined the techniques of the deep neural network with reinforcement learning to detect the adversarial samples of XSS attacks. The detection approach generates adversarial samples and retrains the model to optimize the classification of XSS attacks.

Liu et al. [25] expressed the relationship between words and characters using the word2vec tool released by Google [26]. Liu et al. trained the model using a 2-layer and 6-layer graph convolutional network to detect XSS attacks and showed that accuracy increased with the number of layers in the detection model.

Wang et al. [27] performed experiments on 27000 samples of malicious JavaScript code and trained them using one hidden layer of stacked denoised auto-encoders forming a neural network. The accuracy of detection was 94.82%. However, the approach took longer time for training using the neural network technique. Mokbal et al. [28] designed three models for detecting XSS attacks. Raw data were collected using the first model [28] of random crawling. The second model, built on a neural network, extracted the features from raw data [28]. The third model used artificial neural network (ANN)-based multilayer perceptron (MLP) to classify and predict malicious XSS payloads [28].

### 2.2. Detection of XSS Attacks Using Machine Learning Algorithms

Krishnaveni and Sathiyakumari [10] conducted experiments of 500 Uniform Resource Locators (URLs) by extracting script, applet, and DOM features to classify XSS attack payload using Naïve Bayes, decision tree, and multilayer perceptron techniques. Vishnu and Jevitha [11] extracted 12 features from URL and JavaScript from 43,579 URL instances and classified XSS attack payload using support vector machine (SVM), Naïve Bayes, and decision tree algorithms.

Rathore et al. [29] extracted HTML and URL features from social network services and applied ten machine learning classifiers to detect an XSS attack on webpages of social network sites. The machine learning classifiers were applied on a dataset of 1000 webpages containing 400 malicious and 600 benign webpages collected from XSSed, Alexa, and Elgg sources.

Zhang et al. [30] extracted features from XSS payloads using word2vec and trained the dataset using two unsupervised clustering techniques, Gaussian mixture models (GMMs). Zhang et al. [30] built two GMMs for detecting XSS in web request and web response packets. The XSS payloads are distinguished as two clusters with two different Gaussian functions characterized by the mean and covariance of the data points in a dataset. Any addition of payloads with different feature sets will require retraining to calculate the mean and covariance of all data points again.

### 2.3. Detection of XSS Attacks Using Genetic Algorithm

Khan and Motwani [31] proposed a signature-based intrusion detection of XSS attacks using a genetic algorithm with binary-coded eleven-bit chromosomes. The approach considered the count of suspicious HTML characters and words in the first three bits and the presence of encoded characters in the fifth bit, the following three bits stored the count of suspicious script characters, and the last four bits specified the class of XSS attack.

Suleman and Awan [32] applied genetic algorithm to optimize the machine learning algorithms by reducing the features selected and showed that the accuracy rate of detection of Naïve Bayes, ID3, K-nearest neighbor (KNN), decision tree, and random forest algorithms increased from 76% to 94.99% by applying genetic algorithm for feature selection. Suleman and Awan [32] selected two to eight best features out of 15 features by applying genetic algorithms to detect XSS attacks and optimized detection rate.

Tariq et al. [33] used 30 features proposed by Zhou and Wang [16] and applied a basic genetic algorithm to detect malicious XSS payloads. The model proposed trained dataset on the number of features occurring in the training dataset, and the accuracy rate was as low as 5.78% for a feature “confirm” to 69.60% for feature “script.” Tariq et al. [33] added the reward policy of reinforcement learning to the detection model by updating the novel payloads to the training data to improve the accuracy rate.

### 2.4. Security Testing and Static Code Analysis for XSS Attacks

Gupta and Gupta [7] proposed XSS SAFE, an automated framework for detecting and mitigating XSS attacks. The JavaScript functions were repeatedly injected into the sanitization modules of the source code, and rules were generated to mitigate the attacks. The JavaScript functions and characters were encoded using different encoding techniques and injected to generate detection rules. Gupta and Gupta [7] tested the framework on five Java Server Pages and reported a zero false-positive rate.

Hydara et al. [18] converted the source code of web applications built on Java into control flow graphs and detected the hidden vulnerabilities in control flow paths using genetic algorithms. Taint analysis was done to discover the paths of data insertion through users in five Java-based web applications with 3000 to 80000 lines of code. A genetic algorithm was then used to pass generated XSS input data to identify paths vulnerable to XSS attacks through the input points.

Ahmad and Ali [19] generated XSS test cases using a genetic algorithm and performed dynamic security testing by identifying multiple execution paths in the static code. Ahmad and Ali tested for three types of XSS vulnerability in three small web applications written in PHP. The genetic algorithm's fitness function is minimized to ensure no node is missed in a path testing of a program.

Zhang et al. [21] generated adversarial XSS attacks using the MCTS algorithm and constructed a generative adversarial network (GAN) to detect many XSS payloads.

Jan et al. [24] proposed an automated testing methodology for identifying XML injection attacks. The method randomly generates test data by using five mutation operators. The mutation operators are designed to add randomly selected XML meta-characters, remove quotes from randomly selected attributes, add a closing tag, replicate element, and replace a random element. The method showed that 78.86% of test cases generated bypassed the XML gateway filters.

Duchene et al. [34] detected only reflected XSS (type 1) attacks in web applications by generating malicious input loads using fuzzy inference and genetic algorithms. Duchene et al. [34] considered seven attributes for detecting XSS injection, namely, HTML spaces, attribute delimiter, tag limiter, JavaScript code, URL, and regular text. In their later work, Duchene et al. [35]and Kameleon Fuzz detected and tested for stored XSS attacks (type 2) by generating malicious payloads using fuzzing and genetic algorithm.

Salas and Martins [36] combined penetration testing and fault injection methods to detect XSS vulnerabilities in SOAP UI and XML messages. Salas and Martins [36] showed that the impact of XSS attacks on web services is reduced from 42% to 36% through web service security tokens.

Sahu and Tomar [37] developed a static code analysis tool for assisting web developers in writing secure code. The tool detected hidden code vulnerabilities based on three defensive programming principles. The tool ensures that all unexpected conditions are handled, external data are properly sanitized, and error messages containing technical information are disabled after development.

Liu et al. [38] introduced randomness in the initial payload and the generated high-quality vector using fuzzy inference and operators of genetic algorithm to detect XSS vulnerability in webpages. The attack payloads were generated by replacing and inserting sensitive words, characters, events, functions, blank characters, comments, tags, confusing code, and changing case structure at different location of the XSS attack payload.

### 2.5. Recent Surveys for XSS Attack Process, Detection, and Mitigation in Web Services

In the literature, several techniques are available that perform security testing and detection of input payloads for identifying the existence of unwanted strings and characters in the XML message packet that trigger an XSS attack. Research and review articles about various techniques for security testing and detection of XSS attacks on web services, XML messages, and web applications from the year 2010 to 2022 are studied. Gupta and Gupta [7] presented a survey of ten detection and protection techniques for XSS attacks on the server and browser sides. Gupta and Gupta [7] compared the techniques on five parameters: location of the exploit—server or browser; discovery site; technology for detection; type of XSS attack detected; and modification site—browser, server, or code. Chen et al. [13] discussed the advantages of using machine learning techniques to detect XSS attack. Chen et al. [13] discussed the limitations of whitelisting approach in detection of XSS attack. The library of filtering rule in whitelisting approach is difficult to update with the fast changing nature of XSS attack payload.

The taxonomy of XSS attacks and worms is given by Liu et al. [39]. The authors presented a state-of-the-art process to detect whether a webpage is vulnerable to XSS attack or not. Liu et al. [39] categorized defensive techniques to prevent XSS attacks into black-whitelisting, string injection, static analysis, taint flow analysis, and machine learning [39].

Mishra and Gupta [40] surveyed eleven techniques for detection and mitigation of XSS attacks including the attack via cookie theft. The authors [40] also presented an approach using cryptography techniques to prevent cookie theft through XSS payloads in web services.

Rodriguez et al. [41] analyzed more than fifty review and research articles to analyze various detection and mitigation techniques of XSS attacks. Mitigation techniques for XSS attacks include laboratory activity emulation, simulation, honeypots, IDS Snort, content analysis, rule filters, cache test, text filter, content security policy, string and URL analysis, XML, input validation, filtering patterns, attack modeling, web scanner, black box testing, defensive programming, concolic test, proxy coding of alphabets, cookies analysis, and session authentication [41].

The security of web services, SOAP UI, and XML messages can be validated through testing in the static and dynamic phases [36]. The static phase of fault detection uses code inspection, static vulnerability analysis, or theorem proof without running the system. The dynamic phase of testing searches for vulnerabilities by sending attack payloads in the request or response message. Other forms of commercial and open-source vulnerability scanners (VS) (like HP Web Inspect, BIXSAN, PathCutter, FLAX, SWAP, IBM Rational AppScan, WSDigger, WebScarab) have limited low coverage of existing vulnerabilities and a high percentage of false positives [36, 41].

Sarmah et al. [42] analyzed several XSS filters, e.g., XSS Auditor, XSS Filter, NoScript, IE8, XSS-immune, XBuster, and Rule-Based, in different web browsers. These filters use the techniques of exact or approximate string matching, string comparison, or regular expressions. However, newer and sophisticated XSS payloads and worms like the Facebook XSS worm bypass such filters. Moreover, most ML techniques for detecting malicious XSS payloads cannot detect DOM-based attacks [42].

### 2.6. Comparative Analysis of Recent XSS Detection Approaches

Web applications and other detection techniques undergo frequent changes with increased dependencies in the source code as the newer attack pattern is detected. These frequent changes made in the web application and security mechanisms incorporate new challenges for testing and removing the bugs [43–45]. The existing techniques in the literature require a complete restructuring of the algorithm, changing the source code [46] and retraining of the training dataset [47]. Moreover, with an increase in the size of web applications, usage of code confusion, and dynamic code generation, the various static, dynamic, and hybrid techniques of detection of XSS attacks also become more complicated [39]. The efforts required to detect changing XSS attack payloads increase exponentially.  Table 1 summarizes the techniques and approaches available in the literature.

The available approaches for detecting and classifying cross-site script attacks on web services are deceived with a newer combination of malicious characters, strings, tags, and scripts commonly referred to as adversarial attacks. A fixed number of features limit existing techniques. Further, these techniques perform with lower accuracy and higher complexity as many identifying features in the dataset are added. These models also do not update the dataset with the values of newly discovered features, thereby increasing data with missing values. The problem of missing values in the existing data leads to loss of information, biased learning, and reduced statistical power.

## 3. Preliminaries and Terminologies

In this section, preliminaries and terminologies necessary to understand the proposed approach have been discussed.

The incremental genetic algorithm (IGA) simulates the evolving nature of attack payloads by incorporating mutation and crossover operations in the feature set [47, 48]. Newly discovered features are added in descending order of their classification accuracy to the existing feature set. The payload population with the newly added feature is then evolved using mutation and crossover. The process of mutation and crossover provides an incremental and recursive learning environment that is highly efficient and adaptable to a training set with a large dimensional space with an improved classification rate. The algorithm projects the problem of searching for a solution in a large multidimensional space by projecting it into a single-dimensional space. The algorithm reduces the cost and effort of deriving classification rules.

The IGA consists of a population with a set of chromosomes which in turn are composed of genes. The algorithm works by computing a fitness function, selection of parents, formation of the child using operations of crossover and mutation, eliminating the least fit individual, and including the more fit individual. The process is repeated in a continuously evolving population until a condition is met that terminates the process.

Gene: A gene is a feature that characterizes an attack payload. A set of genes is combined into a string to form a chromosome. A vector *g*  = {*g*_1_*g*_2_*g*_3_,…, *g*_*n*_} is a one-dimensional row vector consisting of all features identifying an injection attack payload.

Chromosomes: A chromosome, often called a “genotype,” is a collection of features or attributes that provide a solution to a problem being solved through a genetic algorithm. A vector *c* *=* {*c*_1_*c*_2_*c*_3_,…, *c*_*n*_} is a one-dimensional row vector that consists of feature value identifying an injection attack payload. The value of a feature may be continuous real values or binary values.

For an attack payload, *A*, *c* *=* {*c*_1_*c*_2_*c*_3_,…, *c*_*n*_} is a chromosome such that

(1) $$\begin{array}{c} \forall g_{i}\in g,\\ c_{i}=x\text{ where }x,\text{ where }R\text{ is a set of real values,}\;\text{if }g_{i}\text{ exists in Population }P\text{,}\\ c_{i}=0,\;\text{otherwise}. \end{array}$$

Population: A population is an array of chromosomes or individuals. As the new features are added, mutation and crossover are performed, and the initial population gets updated and converges to a solution recursively.

(2) $$\begin{array}{c} \text{Population }P=\begin{array}{cccc} c_{11} & c_{12} & \ldots . & c_{1n}\\ c_{21} & c_{22} & \ldots & c_{2n}\\ \ldots & \ldots & \ldots & \ldots \\ c_{i1} & c_{i2} & \ldots & c_{in} \end{array}. \end{array}$$

Mutation: The small deviation in the feature value is termed as “mutation” in the chromosome. The mutation is done by changing the value of one or more features within a chromosome. For an attack payload, A,

(3) $$\begin{array}{c} \text{original chromosome }cx=\left\{c_{1}c_{2}c_{3},\ldots ,c_{n}\right\},\\ \text{mutated chromosome }cy=\left\{c_{1}^{\prime }c_{2}^{\prime }c_{3}^{\prime },\ldots ,c_{n}^{\prime }\right\},\\ \text{where }c^{\prime }\text{ represents changed value for a gene }g_{i}, \end{array}$$

Crossover: The crossover is an operation where features of two-parent chromosomes are swapped to form new child chromosomes. The swapping of features may be done at a single point and multiple points, or in a random manner. The process of crossover generates a new set of child chromosomes.

(4) $$\begin{array}{c} \text{Parent}1\text{ chromosome }cx=\left\{c_{1}c_{2}c_{3}c_{4},\ldots ,c_{n}\right\},\\ \text{Parent}2\text{ chromosome }cy=\left\{c_{1}^{\prime }c_{2}^{\prime }c_{3}^{\prime }c_{4}^{\prime },\ldots ,c_{n}^{\prime }\right\},\\ \text{After crossover at position }2\text{,}\\ \text{Child }1\text{ chromosome }cx^{\prime }=\left\{c_{1}c_{2}c_{3}^{\prime }c_{4}^{\prime },\ldots ,c_{n}^{\prime }\right\},\\ \text{Child }2\text{ chromosome }cy^{\prime }=\left\{c_{1}^{\prime }c_{2}^{\prime }c_{3}c_{4},\ldots ,c_{n}\right\}. \end{array}$$

Adapting to new features: The IGA used for classifying the injection attack vector consists of the formation of the initial population, integration of old and new features, and incremental evolution of new features. The current features are retained in the population, and the newer features are added to the current chromosomes. For more than one feature discovered, the accuracy rate of each of the newly discovered features is calculated and arranged in descending order. The newly discovered features are then appended to the old chromosome ruleset to update the population. A gene set *g*  = {*g*_1_*g*_2_*g*_3 _ *g*_4_,…, *g*_*n*_}, with two newer features *g*_*n*+1, _*g*_*n*+2_, is integrated into the gene set such that *g'* = {*g*_1_*g*_2_*g*_3_ *g*_4_,…, *g*_*in*_*g*_*n*+1, _*g*_*n*+2_} and integrated chromosome *c'* *=* {*c*_1_*c*_2_*c*_3_*c*_4_,…, *c*_*in*_ *c*_*n*+1_ *c*_*n*+2_} where *c*_*n*+1_ and *c*_*n*+2_ represent the values for the new feature *g*_*n*+1, _ and *g*_*n*+2_.

Fitness Function: The fitness of a chromosome is calculated by the percentage of payloads correctly classified by the ruleset of the chromosome [47]. The global fitness of a chromosome is defined in the whole search space. While adding the new features to form a more fit chromosome, the local fitness function considers the newer payload patterns in a local region of search space. The chromosome that correctly classifies highest number of payloads in a dataset is selected for defining the rules. Such chromosomes have highest fitness function value.

(5) $$\begin{array}{c} \text{Global Fitness function f}-\text{global}:\frac{\text{Number of Correctly classified payloads}}{\text{Total Number of payloads in the dataset}}\\ \text{Local Fitness function f}-\text{local}:\frac{\text{Number of Correctly classified newer payloads }}{\text{Total Number of newer payloads}}. \end{array}$$

Termination Criteria: The evolution process is stopped if any one of the following four criteria is reached:

- Maximum generation limit of the population is attained.
- The best fitness of the chromosome is obtained.
- There is no improvement in the accuracy and fitness of the best chromosome after a specified number of generations.
- Performance of validating the data is less than 10% for the last 20 generations.

Classification Mechanism: The IGA is a rule-based classification technique that assigns each instance of a payload to a class by identifying relationships among features in it. The rules are represented in the form of IF (Condition 1) & (Condition 2) &…. Condition *n*-THEN (Class *A*_i_). Each rule has one or more conditions as the antecedent, and a class is identified as the consequent. The conditions are represented as if-then rules, and the values of the set of features are represented in binary form. A ruleset contains several rules, providing a solution for a classification problem. A rule defining a class is represented as follows:

(6) $$\begin{array}{c} R_{i}:If\left(V_{1\_\text{min}}\leq x_{1}\leq V_{1_{\text{max}}}\right)\wedge If\left(V_{2\_\text{min}}\leq x_{2}\leq V_{2\_\text{max}}\right)\ldots \wedge If\left(V_{n\_\text{min}}\leq x_{n}\leq V_{n_{\text{max}}}\right)\\ \text{THEN }y\in C, \end{array}$$

where *R*_*i*_ is a rule label, *n* is the number of features, *x*_1 _*x*_2_,…, *x*_*n*_ is the feature set, *C*  is a class identified for payload *y*, and *V*_*i*_min_ and *V*_*i*_max_ are the minimum and maximum bounds of the feature *x*_*i*_, respectively [47, 48].

## 4. Research Methodology

**Figure 2:**
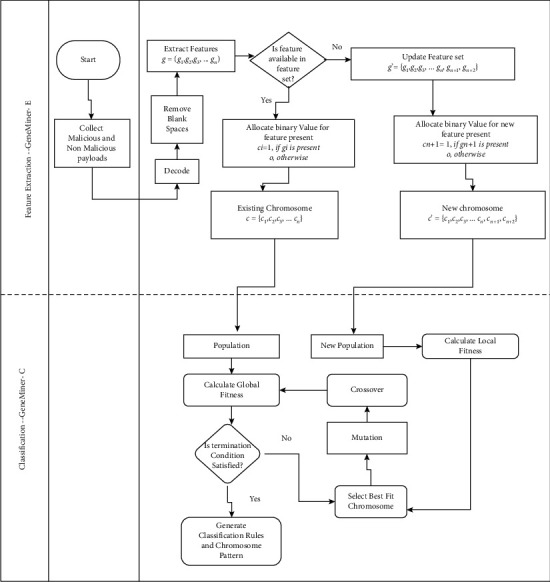
Proposed approach GeneMiner for detection of XSS attacks.

This section discusses the proposed GeneMiner approach to detect malicious payloads triggering XSS attacks, as shown in Figure 2. The authors have collected the XSS payloads from various open-source repositories to conduct the experiments. The payload data are preprocessed, features are extracted, and the existence of a feature in the attack vector is populated in a database using the GeneMiner-E extraction model. The experiments are conducted to classify the payload as malicious or nonmalicious using the GeneMiner-C classification model.

### 4.1. Dataset Collection

The authors collected the payloads from various sources for conducting the experiments to detect XSS vulnerability. The dataset contains the 6606 malicious records collected from https://github.com/payloadbox/xss-payload-list/, and 18,151 malicious and 1,35,507 nonmalicious records from https://github.com/duoergun0729/1book/tree/master/data. In total, training dataset contains 160, 264 URLs with payloads that may trigger an XSS attack. The testing dataset in the experiment is gathered from GitHub sources and security forums, which consists of 3497 malicious and 6503 nonmalicious URLs provided by Zhou and Wang [16].

### 4.2. GeneMiner-E: Data Preprocessing, Decoding, and Feature Extraction

For preprocessing of the data and extraction of features from the data, an approach, GeneMiner-E, has been proposed. The GeneMiner-E anonymizes the data by removing the domain part of the URLs and websites. Then, it decodes the encoded characters available in the payloads and cleans the data by removing blank spaces. For normalizing the data, the approach extracts the features from the payloads and prepares the final dataset by marking the feature set available in the attack vector as one and zero for the nonavailable feature.

The XSS payloads have common features of the same sensitive words and characters but differ in triggering XSS attacks. The payloads also contain characters obfuscated using encoding mechanisms such as URL encoding, ASCII encoding, Unicode escaping, and hexadecimal encoding. These characters are decoded to original characters. For example, an attack payload may have a left-angle bracket < in hexadecimal code as %3C. The attack payload with characters *'albr/er&nbsp;&ensp;&emsp* bypassed through filters as the keyword “alert” has been modified by inserting a break tag (br/ and variants of blank space, namely, single space (&nbsp;), double-space (&ensp;), four space (&emsp;).

A payload from client to server or server to the client is classified into two classes: vulnerable and nonvulnerable. An attacker may modify the payload by injecting malicious characters, words, scripts, tags, or codes. All such malformed payloads capable of triggering an XSS attack on a webpage are labeled as malicious payloads, while the payloads that do not trigger an XSS attack are nonmalicious.

XSS payloads are identified by sensitive charact*'albr/er&nbsp;&ensp;&emsp*, keywords, HTML tags, scripts, redirection links, and unusual lengths of data [16, 33]. A few sensitive words and characters are alert, prompt, script, angle brackets, parenthesis, and onmouseover. The decoder module of GeneMiner also identifies the obfuscated URL modified through various encoding mechanisms, Unicode escape, inserting blank spaces, inserting invalid characters, case mixture, and other forms of malicious attack payloads [21]. The existence of these words and characters in a payload is considered a feature as it defines the characteristic nature of malicious payloads. For conducting experiments, 40 features are identified in the dataset of 1,64,204 records.  Table 2 shows the list of 40 features extracted from the collected XSS payloads using GeneMiner.

### 4.3. GeneMiner-C: Classification Model

The training set of malicious and nonmalicious XSS payload data forms the initial population in the proposed approach. The dataset contains unique features and independent variables, while the class identified is the dependent variable. The authors constructed two datasets: one training and another testing dataset. The classification model is constructed using an incremental genetic algorithm on the training dataset and validated using the testing dataset.

The population is a binary matrix of order *m*  ×  *n*, where *m* is the number of URLs and *n* is the number of features called genes extracted from the dataset. Every row of the population matrix consists of chromosomes representing the payload as defined in

(7) $$\begin{array}{c} c=\left\{c_{1}c_{2}c_{3}\ldots .c_{n}\right\}\text{ is a chromosome such that}\\ \forall g_{i}\in \text{gene},g,\\ c_{i}=1\text{ if }g_{i}\text{ exists in }P\text{ and,}\\ c_{i}=0,\text{otherwise}. \end{array}$$

**Figure 3:**
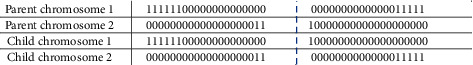
Single-point crossover.

The combinations of attack payloads are generated by using a single-point crossover. The vertical dotted line in Figure 3 represents a single-point crossover. The process of mutation and crossover generates a unique and large number of attack payloads that provides a solution to classify the payload as vulnerable or nonvulnerable.

On identifying a newer attack payload, the newly discovered features are appended to the chromosomes of the population matrix. For example, consider a payload city = test%3Cscript%3Eale%20rt%28/42873/%29%3 C/script%3E. The features extracted from the payload using the proposed GeneMiner-E are “script, left-angle-bracket, alert, open-parenthesis, close-parenthesis, right-angle-bracket.” Initially, there are six features, and the chromosome corresponding to the payload will have these features marked as “1.” With the addition of the new attack payload with the new feature “prompt,” the number of features is increased from 06 to 07. Similarly, with the discovery of two new features, “onload” and “onmouseover,” in the attack payload, the chromosome is modified by integrating the newly discovered features, as shown in Table 3. The absence of the new feature in the old payload is given a binary value of 0, and new features are appended to the modified chromosome with a value of 1.

The process of mutation and crossover continues till the termination condition is reached. A threshold of the number of generations and rules is set as a termination condition. A classification rule is obtained for each generation that classifies a certain number of chromosomes. For each classification rule, the local fitness and global fitness are calculated. The rule with the highest fitness function value, correctly classified instances, is further used in the training model to classify attack payloads. For evaluating the proposed GeneMiner, the threshold for termination condition of the proposed classification scheme has been kept at 200 generations and 100 rules by mutating one gene and single-point crossover for maximum classification rate [48].

## 5. Experiments and Results

The experimental environment used in this paper was run under Windows 11 operating system, equipped with a 2.6 GHz 6-core Intel Core-i7 processor and 16 GB RAM. In the experiments, the effect of adding new features and a new adversarial attack vector on the classification efficiency is analyzed on the proposed GeneMiner approach. Initially, the training dataset consists of twenty features with 44000 malicious and nonmalicious records. Newly discovered payloads are added to the initial training set in a newly discovered five feature batches. The classification accuracy of the proposed approach is compared with the popular classification techniques, i.e., Naïve Bayes (NB), random forest (RF), logistic regression (LR), support vector machine (SVM), AdaBoost, and multilayer perceptron (MLP). The results obtained are presented in Table 4.

The performance of the proposed GeneMiner is further evaluated using the accuracy, sensitivity, specificity, precision, and *F*-score metrics as defined in equations (8)–(12). The results obtained for the feature set are reported in Table 5.

(8) $$\begin{array}{c} \text{Accuracy}=\frac{\text{TP}+\text{TN}}{\text{TP}+\text{FP}+\text{TN}+\text{FN}}\;\times \;100, \end{array}$$

(9) $$\begin{array}{c} \text{Sensitivity}=\frac{\text{TP}}{\text{TP}+\text{FN}}\;\times \;100, \end{array}$$

(10) $$\begin{array}{c} \text{Specificity}=\frac{\text{TN}}{\text{FP}+\text{TN}}\;\times \;100, \end{array}$$

(11) $$\begin{array}{c} \text{Precision}=\frac{\text{TP}}{\text{TP}+\text{FP}}\;\times \;100, \end{array}$$

(12) $$\begin{array}{c} F-\text{Score}=2\;\times \frac{\text{Precision}\times \text{Sensitivity}}{\text{Precision}+\text{Sensitivity}}\;\times \;100, \end{array}$$

where TP, TN, FP, and FN are the number of true positives, true negatives, false positives, and false negatives, respectively.

The set of experiments conducted shows that the addition of a new feature improves the accuracy of detection of XSS attack payload using the proposed GeneMiner approach. The proposed approach has an accuracy of 98.07%, while other classifiers have accuracy as low as 69.49 for the random forest, 89.05 for Naïve Bayes, and 93.7 for logistic regression. Logistic regression and Naïve Bayes approaches are unbalanced in classifying one class. These approaches classify negative classes more accurately with higher sensitivity, but positive classes are identified with lower accuracy. The accuracy, specificity, sensitivity, and *F*-Score of the proposed GeneMiner approach are high, signifying the accurate and balanced classification of vulnerable and nonvulnerable classes.

## 6. Empirical Evaluation with Existing Approaches

The proposed approach for the classification of payloads triggering an XSS attack is compared with the existing approaches available in the literature. The authors of [18, 19, 21, 34, 35] performed security testing using fuzzy evolutionary inference to identify XSS attacks in web applications. Khan and Motwani [31] and Suleman and Awan [32] applied a genetic algorithm for feature selection during the XSS detection methodology. Fang et al. [14, 20] performed decoding of XSS payloads and applied neural network techniques to identify malicious XSS payloads. Lei et al. [15] used the LSTM model for XSS detection, and Melicher et al. [17] applied deep neural network for detecting DOM-based XSS attacks. Liu et al. [25] applied graph convolution networks, and Abimov and Bianchi [49] used convolutional deep neural network (CNN) for the detection of XSS attacks. Zhou et al. [21, 30] applied machine learning techniques, [33] applied a genetic algorithm along with reinforcement learning to detect XSS attacks, and [50] detected reflected XSS attacks using reinforced learning. It is observed that only 06 out of 16 articles focused on evolutionary algorithms for the detection of XSS attacks, while only 01 focused on XML injection [51, 52].

The existing approaches of [14, 21, 33, 42] detect XSS payload by extracting script tags, special characters, eval function, features from URLs, and cookies but do not consider CSS style, dangling markups, and polygons. It is observed by [53] that the escape rate of XSS payloads from various detection models of machine learning and deep learning has reached 85%, and such techniques are less efficient for the detection of adversarial attacks. The proposed GeneMiner approach has extended the feature set by incorporating CSS style, dangling markups, encoded characters, and polyglots to detect malicious XSS payloads in XML messages.  Table 6 shows the classification accuracy using the proposed approach and the existing approaches.

The existing approaches of XSS attack detection using generative adversarial network [30] have an accuracy rate of 94.59%, and enforcement learning [21] reaches an accuracy up to 97.78, using deep neural network [20] (with an accuracy of 91.7%). Other machine learning techniques detect XSS payloads with less than 90% accuracy. The proposed GeneMiner approach has reached the accuracy of 98.5 with 40 features detecting varied, changing, and evolving malicious XSS payloads. The existing approaches available in the literature do not evolve to changing nature and addition of newer features of XSS in attack payloads. Due to computational complexity, these approaches are also not suitable with higher dimensional feature sets. The proposed GeneMiner approach evolves itself to the modifying nature of XSS attack payloads and updates the training set incrementally with the addition of newer features. The proposed GeneMiner approach also optimizes the search space by reducing it to single-dimensional search space.

## 7. Conclusion and Future Work

The ever-increasing and varying nature of XSS attack payloads bypasses the filtering mechanisms of existing techniques. The XSS attack results in defacing websites, destruction of data, disruption of services, and disclosure of confidential data in web applications. This paper proposes a GeneMiner approach to detect XSS attacks in XML documents. The proposed approach evolves itself to detect malicious payloads consisting of newer functions, features, combinations, encoded characters, and obfuscation techniques. The experiments were conducted by collecting open-source payloads and training them to detect XSS attack payloads. The proposed approach works in two steps. In the first step, the features that define the XSS attack are extracted from many payloads using the GeneMiner-E model. The newer features identified in newer malicious payloads are appended to the existing feature set. In the second step, the GeneMiner-C creates a single-dimensional ruleset to classify attack payloads. The experimental results showed that the proposed approach identifies the new adversarial attack payloads and gives a higher accuracy of 98.5%, which is better than the existing classification approaches. In the future, the authors intend to extend the proposed approach for detecting other forms of injection attacks on web services.

## Figures and Tables

**Table 1:** Summarized limitation of recent XSS detection and testing approaches.

Reference	Number of features	Type of XSS attack detected	Number of malicious instances in dataset	Approach	Limitations

[10]	04	R,S,D	500	ML	The dataset tested is very low in number.
[11]	12	R,S,D	43,579	ML	URL and script features were extracted. Polyglots and other complex features were not considered. Decoding of URLs was not considered
[14]	07	R,S,D	33,426	LSTM, DL	XSS attacks due to polyglots, angular JS, and CSS-style tags could not be detected. The detection algorithm lacks optimization as the number of features increases
[15]	07	R,S,D	32,168	LSTM, RNN	
[20]	07	Adversarial	33,426	LSTM, DNN, RL	
[28]	41	R, S	38,569	MLP	
[29]	25	R, S	400	ML	
[30]	10	R	35,884	GMM	DoM and stored XSS attacks were not explored
[16]	30	R,S,D	16,151	Bayesian network and EL	Attacks due to polyglots, JS, file upload, and CSS could not be detected
[17]	10	D	1,80,000	DNN	Reflected and stored XSS was not detected
[25]	07	R, S	5000	GCN	DOM type of XSS attack could not be detected
[31]	02	R,S,D	—	Signature and entropy	The detection is based on several script and URL features present in the payload. The exact pattern, sequence, and combination are not considered
[32]	15	R	11054	GA for feature selection and ML for detection	Only URL-based features were selected to detect website phishing attacks
[33]	30	R,S,D	50540	GA and RL for increasing accuracy	Polyglots and other complex XSS payloads could not be detected. Benign payloads in the dataset are considered devoid of all 30 features, while in the actual scenario, an input payload may have a few features but still not malicious. The accuracy was as low as 76% using GA, which increased after applying RL
[34, 35]	08	R, S	—	FL and GA	Attack grammar requires manual updating of the feature set. Very few attack grammar rules are generated, limiting fault detection
[27]	15	R, S	14783	Stacked denoised auto-encoder DNN	Only script tags and functions triggering XSS attacks were considered. Long training time is another limitation
[42]	21	R, S	289	ML	DOM-based XSS attack could not be detected. The training set consists of a low number of malicious instances

R: reflected XSS; S: stored XSS; D: document object model XSS; DL: deep learning; ML: machine learning; FL: fuzzy logic; RL: reinforcement learning; GA: genetic algorithm; RNN: recurrent neural network; MLP: multilayer perceptron; DNN: deep neural network; EL: ensemble learning.

**Table 2:** Features extracted for detection of XSS payload using proposed GeneMiner.

Length	Onfocus	Semicolon	Window_x	Click

Closeparen	Evalfunc	Src	Onerror	Onkeydown
Openparen	This	If_func	Sq	Onkeyup
Alert	Doc_cookie	Img	Href	Onkeypress
Close angle	Replacefunc	Onmouseover	URL	String_dot
Open angle	Try	HTTP	Prompt	Fromcharcode
Script	Catchfunc	endif_func	Iframe	Reload
Dq	expressionfunc	Formation	Change	Filters

**Table 3:** Incremental updating of feature set and chromosomes using GeneMiner-E.

S.no	Attack payload	Decoded	# features	Features extracted	Initial chromosome (*c*_*n*_)	Modified chromosome (*c′* *=* *c*_*n* *+* 1_)	Modified chromosome *c*″ *=* (*c*_*n* *+* 3_)

1	city = test%3Cscript%3Eale%20rt%28/42873/%29%3C/script%3E	city = test<script>alert(42873) </script>	06	script, <, alert, ( ) >	111 111	111 111 **0**	111 111 **000**
2	city = test%3Cscript%3Eprom%20pt%28/42873/%29%3C/script%3E.	city = test<script>prompt(42873) </script>	07	script, <, ( ),>,prompt	—	101 111 **1** (prompt)	101 111 **100**Absence of newly discovered features is marked as 0
3	<sCRipT < svg/onload = *′*+″/+/onmouseover = 1/ + /[∗// + alert(1)//*′*>	<script < svg/onload = onmouseover (alert1)>	09	script, <, ( )>,onload, onmouseover	—	—	111 111 011 (onload, onmouseover)

**Table 4:** Impact of the addition of new features on accuracy of GeneMiner and other approaches.

Number of features	Naïve Bayes	RF	LR	SVM	AdaBoost	MLP	Proposed GeneMiner

20	59.25	68.41	68.48	68.48	66.48	68.48	80.73
25	66.8	64.1	64.3	69.54	68.61	69.47	91.38
30	81.6	73.7	76.1	73.19	68.61	77.30	91.45
35	88.78	69.61	98.1	87.39	87.61	87.21	96.26
40	89.05	69.47	93.76	88.98	93.49	96.80	98.50

**Table 5:** Comparison of classification accuracy of GeneMiner with other approaches on the addition of newer attack payloads.

Approach	Accuracy	Sensitivity	Specificity	Precision	*F*-Score

Naïve Bayes	89.05	96.44	85.3	76.88	82.52
Random forest	69.47	44.57	82.1	55.8	49.56
Logistic regression	93.76	98.02	91.6	85.5	89.46
SVM	88.98	89.01	79.01	90.60	89.2
AdaBoost	93.49	93.50	91.07	94.10	93.30
MLP	96.80	95.29	95.29	96.80	96.30
Proposed GeneMiner	98.50	96.25	98.99	97.99	98.03

**Table 6:** Empirical comparison of GeneMiner with existing approaches.

GeneMiner proposed	GAN [21]	EL [16]	RL [20]	RF [29]	DL [27]	CNN [49]	NB [16]	SVM [16]	LR [16]	RF [16]

98.5	94.59	97.78	91.7	97.2	94.82	90.2	84.44	85.76	85.82	85.76

GAN: generative adversarial network; EL: ensemble learning; RL: reinforced learning; DL: deep learning; CNN: convolution deep neural network; NB: Naïve Bayes; SVM: support vector machine; LR: logistic regression; RF: random forest.

## Data Availability

The dataset contains the 6606 malicious records collected from (1) https://github.com/payloadbox/xss-payload-list/, and 18,151 malicious and 1,35,507 nonmalicious records from (2) https://github.com/duoergun0729/1book/tree/master/data. The testing dataset in the experiment is gathered from GitHub sources and security forums, which consists of 3497 malicious and 6503 nonmalicious URLs provided by the authors of [16] Zhou, Y., amp; Wang, P. (2019).
